# Evaluation and integration of cancer gene classifiers: identification and ranking of plausible drivers

**DOI:** 10.1038/srep10204

**Published:** 2015-05-11

**Authors:** Yang Liu, Feng Tian, Zhenjun Hu, Charles DeLisi

**Affiliations:** 1 Bioinformatics Graduate Program, and Department of Biomedical Engineering, Boston. University, 24 Cummington Mall, Boston, MA 02215, USA

## Abstract

The number of mutated genes in cancer cells is far larger than the number of mutations that drive cancer. The difficulty this creates for identifying relevant alterations has stimulated the development of various computational approaches to distinguishing drivers from bystanders. We develop and apply an ensemble classifier (EC) machine learning method, which integrates 10 classifiers that are publically available, and apply it to breast and ovarian cancer. In particular we find the following: (1) Using both standard and non-standard metrics, EC almost always outperforms single method classifiers, often by wide margins. (2) Of the 50 highest ranked genes for breast (ovarian) cancer, 34 (30) are associated with other cancers in either the OMIM, CGC or NCG database (*P* < 10^−22^). (3) Another 10, for both breast and ovarian cancer, have been identified by GWAS studies. (4) Several of the remaining genes--including a protein kinase that regulates the Fra-1 transcription factor which is overexpressed in ER negative breast cancer cells; and Fyn, which is overexpressed in pancreatic and prostate cancer, among others--are biologically plausible. Biological implications are briefly discussed. Source codes and detailed results are available at http://www.visantnet.org/misi/driver_integration.zip.

The identification of aberrant genes that alter cellular processes and thereby drive transformation, is among the most critical challenges in cancer biology^1^. There is no shortage of candidate genes or alterations: high throughput sequencing^2^,^3^ has uncovered more than a million of mutations, and the number is growing rapidly. Most of these are, however, passengers, conferring no fitness advantage on the tumor^4^,^5^ - and those that do, may not be seen frequently enough to be readily distinguishable from background mutations. Because the number of candidates is very large, and the expected number of targets is relatively small, computational screening methods have become an important component of the search for drivers.

Not surprisingly, a number of methods have been developed, these falling into two main categories: gene level and module level. The gene level methods use mutation (frequency and tissue distribution) to make a statistical decision to classify a gene as a driver rather than a passenger. These approaches assume that driver genes independently confer a selective advantage on tumor initiation and progression, and that they can be identified by statistically significant attributes. The most common approach in this category identifies mutated genes that occur at unusually high frequency across a wide range of tumor samples^6^,^7^,^8^. Other methods identify genes that have a large number of functional variants associated with transformation^9^; or that have clustered mutations^10^. Exploiting the over-representation of mutations in protein phosphosites or protein kinase domains has also been effective^11^.

Although gene level approaches have helped to identify numerous driver gene candidates, like all methods, they have limitations. Since mutations are large in number and diverse in type, the frequency of any particular mutation pattern across a set of samples is low. This makes statistical distinctions and reproducibility across different populations difficult to establish. In addition, genes seldom work alone, but instead generally cooperate to trigger phenotypic change.

The second category of methods, based on modules, exploits the idea that subsets of cancer causing genes subserve similar functions and interact strongly. Consequently they don’t necessarily rely on mutations to infer candidates, and in principal can identify potential drivers even when mutation frequencies are too rare to be detected by gene-based methods.

Some module-based methods identify candidates by evaluating metrics that define their linkage to known cancer genes^12^. Others identify relevant gene sets by maximizing modularity based on either a Functional Linkage Network (FLN) (Huang *et al*, http://visantst.bu.edu:8080/) or a Human Interaction Network (HIN)^13^. Another widely applied method utilizes mutual exclusivity^14^,^15^ to systematically identify oncogenic modules. Module-based approaches usually integrate multiple data types including, among others, expression data, CNV data and functional similarity from distinct networks (Functional Linkage Network, Human Interaction Network, KEGG pathway etc.). This improves statistical power, and the consistency of predictions^16^. However, since all known drivers (genes identified as drivers in Cancer Gene Census (CGC) or the Online Mendelian Inheritance in Man (OMIM)) are identified solely on the basis of mutations (frequency and tissue distribution), module based decision criteria are less direct than the single gene methods based on mutation.

In addition to using methods independently, some attempts have been made to use more than one classifier by requiring that at least two agree in order to classify a gene as a driver^17^,^18^. The philosophy of using more than a single method is similar to ours, but the procedure differs substantially from machine learning approaches, which integrate methods and make assignments in a principled manner, as we now explain.

Machine learning (multivariate statistical) methods, have been widely applied in many areas of inquiry including biomedical science^19^,^20^,^21^,^22^, and invariably provide better performance than single feature classifiers. In effect, they all attempt to find an optimal boundary that separates categories such as tumor subtypes^23^ or protein binding sites^24^. It is noteworthy that finding an optimal boundary during training, and using it to make decisions, removes the arbitrariness of simple decision criteria that are used in both module and gene based methods. This allows an unambiguous assessment of true and false positive rates by cross validation. Such rates are not obtainable using decision thresholds, since the number of true and false positives will depend on where the threshold is set.

All machine learning methods begin with a vector of features, which takes on different values for each member of the two categories. For example, the separation of tumor subtypes might begin with the expression levels of a select set of human genes as the features^19^, so that each sample is characterized by a particular vector of expression levels. If there are *m* samples and *n* features, an appropriate multivariate (machine learning) method would be used to find an optimal boundary separating the samples in an *n* dimensional space. In general, the higher the dimensionality (i.e., the larger the number of features), the better the separation^24^,^25^. Thus separation based on multiple features, will almost always be more effective than separation based on a single feature, subject to the usual over-fitting caveat.

Here we formulate an ensemble classifier (EC) and apply it to the discovery of driver candidates in breast and ovarian cancer samples from the Cancer Genome Atlas (TCGA)^26^,^27^. We take as our definition of cancer drivers, mutated genes that have been classified as cancer causing in either the Cancer Gene Census (CGC) ( http://cancer.sanger.ac.uk/cancergenome/projects/census/), or the Online Mendelian Inheritance in Man (OMIM) ( http://www.omim.org/).

We compared the top 50 genes determined by EC (EC50), with the Top 50 genes identified by each of the 10 methods by two different criteria for breast and ovarian cancer. We find that EC ranks first or is tied for first, by both criteria, for both cancer types, and that its predictive power is more stable than that of the individual methods.

We also calculated the extent to which the top 50 predictions by each method was enriched in cancer associated genes from COSMIC, OMIM and the Network of Cancer Genes (NCG) ( http://ncg.kcl.ac.uk/)^28^. For the individual methods, the enrichments, or positive predictive values (PPV) for breast cancer ranged from 12–58% (average 37.4%) compared to 68% (34/50) for EC. For ovarian cancer, the PPVs ranged from 4–64% (average 36.2%) compared to 60% for EC (30/50). The PPV of 64%, slightly higher than that of EC, was achieved by the FLN and NetBox.

We find that of 10 of the remaining 16 breast cancer EC50 genes and 10 of the remaining 20 ovarian cancer EC50 genes that are not annotated as cancer associated, have records in either the GWAS Catalog^29^ or the Genome Association Database (GAD)^30^. Consequently 6 (10) genes have not been previously associated with breast (ovarian) cancer in any large scale population studies.

The performance of the method, the high degree of enrichment, and the biological evidence, as indicated in the discussion, suggest that the predicted candidates are plausible, and that they should be considered high priority targets for epidemiological validation.

## Results

**Figure 1:**
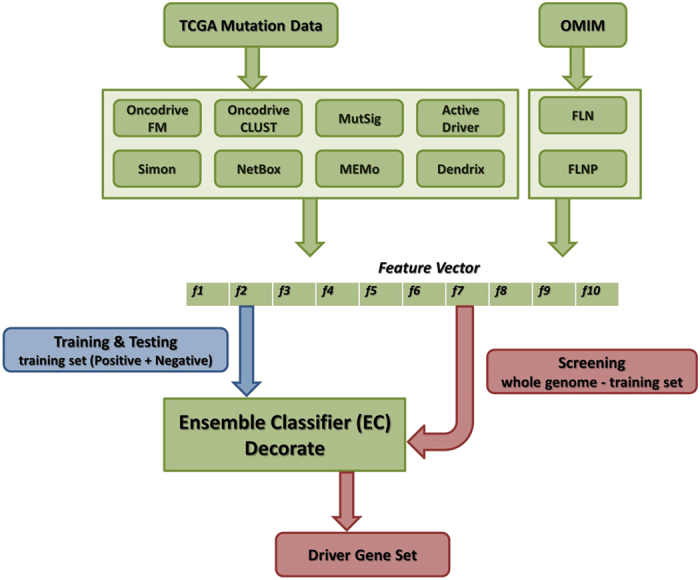
Ensemble classifier (EC) flow chart. TCGA mutation data is used as input to 8 of the 10 publicly available classifiers; two of the module methods take OMIM data as input. EC is applied to the training set (Methods) as part of a ten-fold cross validation procedure, to obtain driver/passenger outputs. The vectors are separated in a ten dimensional space by the Decorate ensemble classifier. After training and cross validation, all known human genes, except those used for training, are scored.

Details of the algorithm are described in Methods. Briefly, method integration is achieved by separating drivers from passengers in a 10 dimensional space, where points are vectors whose elements are the values of 10 individual methods. Positive (known drivers) and negative (putative passengers) training sets were selected as described below, and extracted for use with the DECORATE (Diverse Ensemble Creation by Oppositional Relabeling of Artificial Training Examples)^31^ ensemble classifier. After 10-fold cross validation, the classifier was applied to all genes in protein coding regions except those used for training. We also applied the 10 publicly implemented methods individually (Fig. 1, Table 1) to obtain a reference set of predictions against which to assess the ensemble classifier.

All protein coding regions in the human genome, except those used for training, were ranked by the ensemble classifier as well as by the individual classifiers. We focus on the Top 50 genes generated by each method.

### Breast Cancer

#### Performance

The true positive (TP) and true negative (TN) rates for the ensemble classifier (EC) were estimated at 0.65, 0.98, respectively, as described in Methods. The true negative rate (specificity) is included for completeness, but it is important to note that it is not informative. As indicated in methods, because the number of drivers is small, the chance that a negative gene will be assigned to the positive set is extremely small.

**Figure 2:**
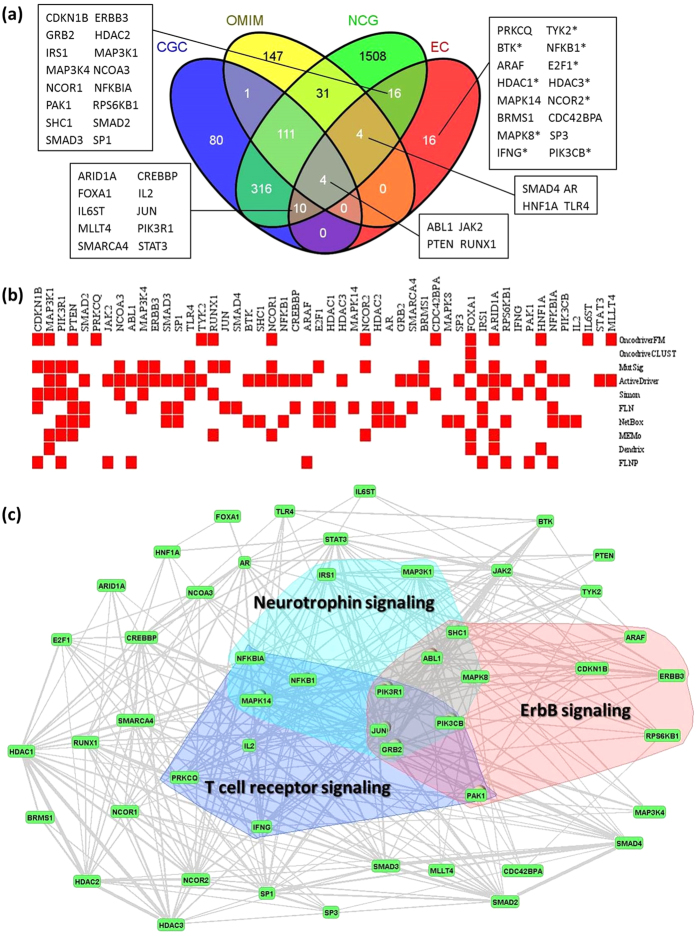
Ensemble predictions for breast cancer. (**a**) Thirty-four of the top 50 genes selected by EC (EC50) are either in CGC, OMIM, or NCG. The Venn diagram displays their distribution among the three databases. Of the remaining 16 genes, 10 have been discovered in GWAS studies (indicated by asterisk). (**b**) EC50 genes identified by the 10 independent classifiers. (**c**) EC50 genes and enriched signalling pathways mapped onto the FLN as explained in the text. Only links with weights greater than 0.1 are retained.

We determined enrichment of cancer genes in the top 50 predictions (PPV) by testing genes that are annotated as cancer related in either CGC, OMIM or NCG (Fig. 2a), including those that have not yet been definitively classified as drivers, but excluding, as usual genes in the training set. We obtained a PPV (true positives/number of calls) of 34/50 = 0.68.

Of the 16 genes that were not classified as positive, ten (marked as asterisk in Fig. 2a) have cancer related records in either the GWAS Catalog^29^ or GAD^30^. The remaining 6: *PRKCQ, ARAF, MAPK14, BRMS1, CDC42BPA, SP3*, have not been confirmed in any large scale clinical studies and are considered predictions. The extent to which the individual methods identify these genes is shown in Fig. 2b.

#### Intergenic relations

Most cancers have complex genotypes. We took two approaches to identifying genes that might contribute to the same cancer, either alone, or in combination with other genes. (i) We used the Fisher exact test to identify KEGG pathways ( http://www.genome.jp/kegg/pathway.html) that might be statistically enriched in EC50 genes compared to the human genome background using DAVID^32^. We found 17 (*FDR* < 0.01) such pathways (Table 2). (2) We overlaid the pathways on a functional linkage network (FLN)^12^. An FLN is a network of nodes (representing genes) connected in such a way that functionally related genes are in proximity to one another, with connections weighted in a principled manner by multiple sources of evidence^12^. Figure 2c is a VisANT^33^ display of the relation, on an FLN, between the three most significantly enriched signaling pathways: ErbB signaling, T-cell receptor signaling and Neurotrophin signaling.

#### Predictive performance compared to individual classifiers

The 10 independent classifiers fall into two categories: statistically based and module based. We took as candidates, genes with *P* < 0.05 using the former method, and genes within a module, using the latter. Unfortunately the original publications that introduced these methods contain very little information on the true positive and true negative rates 7,8,9,10,11. In most cases this is undoubtedly because they use simple thresholds, so there is no well-defined number of true positives.

Although true positive rates for threshold based methods depend on where the threshold is set, we can look at performance in a slightly different way, by calculating the true positive and true negative rates for cancer associated genes (i.e. cancer related in either CGC, OMIM or NCG) in the top 50 of each of the 10 methods. This gives what we will refer to as sensitivity and specificity surrogates, to stress that they are not obtained the same way as the sensitivity and specificity is obtained using a training procedure, which finds an optimal boundary, and therefore doesn’t depend on threshold adjustment. The surrogate specificities were, as with EC, statistically indistinguishable from 1, again, not informative. The surrogate sensitivities ranged from 3-30%. These numbers are useful for comparing the 10 methods with one another, but they are not useful for comparison with EC.

**Figure 3:**
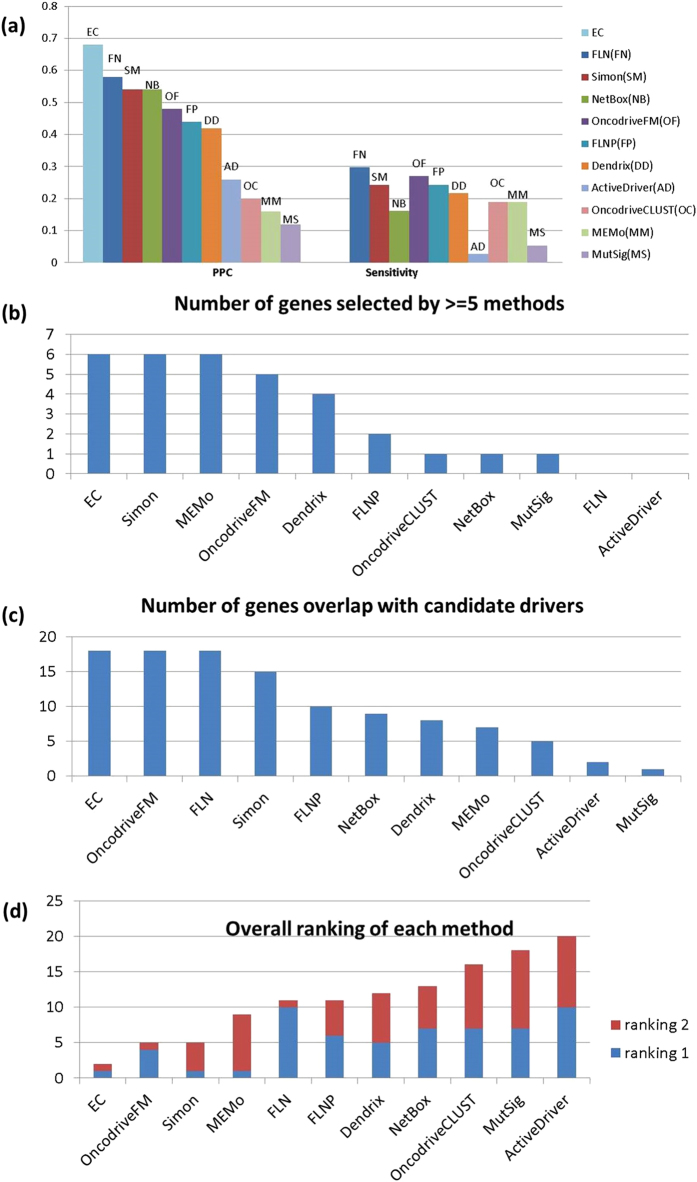
Comparison of performance metrics for the ensemble classifier and single feature classifiers for breast cancer. (**a**) Sensitivity and PPV for each of the methods. (**b**) The number of genes in Top 50 that are identified by at least 5 methods. No genes can be selected by more than 5 methods in FLN and ActiveDriver. (**c**) The number of genes in Top 50 that are annotated in two breast cancer studies. (**d**) Overall ranking of each method based on the sum of rankings in (**b**) and (**c**).

Unlike sensitivity and specificity, the enrichment of cancer associated genes in the top 50 is unambiguously estimated for all methods. Enrichment scores (PPV) were determined in the same way they were for EC. The PPVs for the individual methods ranged from 12-58% (average 37.4%), compared to 68% for EC. The results are summarized in Fig. 3a.

We also used two additional criteria to compare performance. For each method we counted the number of genes in the Top 50 that (i) are identified by at least 5 individual methods (i.e. at least half the methods); and (ii) appear in two other well-known breast cancer studies^26^,^34^, and would therefore be considered candidate drivers (a total of 216). Each method is ranked by these criteria, and an overall rank was assigned using the sum of the two ranks. Although a number of the methods do as well as EC in one or the other of the criteria (Fig. 3b,c), their performance is less stable. In particular, EC is tied for first place by both criteria, giving an overall rank of 2, somewhat higher than OncodriveFM, which placed second with a rank score of 5 (Fig. 3d). The high standing of EC by all 3 criteria supports the idea that the reliability is more stable than that of any other method.

### Ovarian cancer

#### Performance

**Figure 4:**
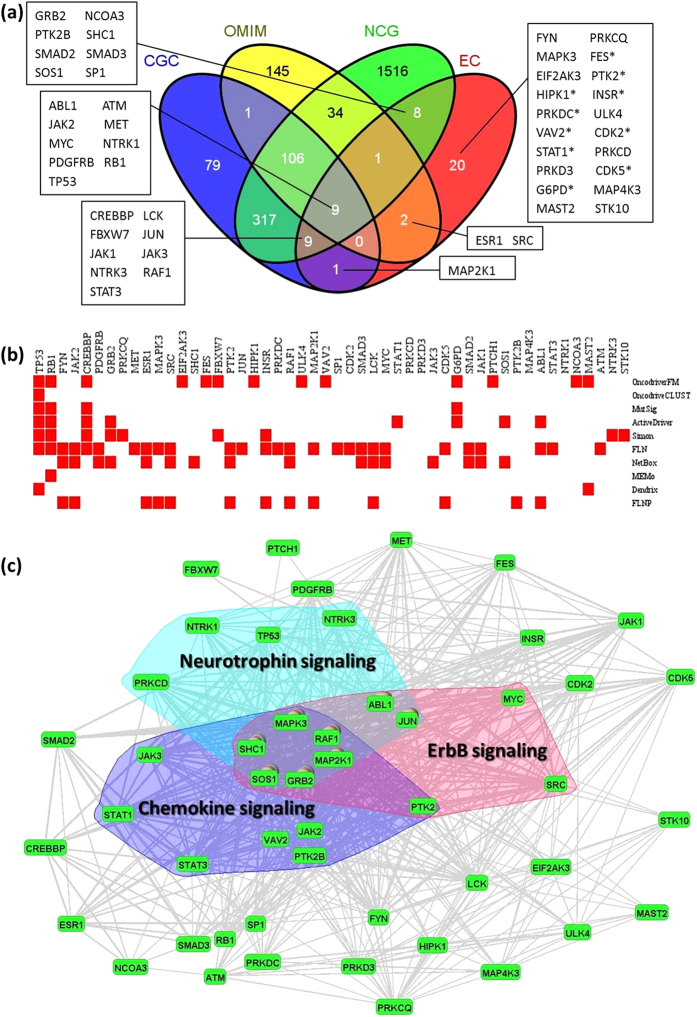
Ensemble predictions for ovarian cancer. (**a**) Thirty of the top 50 genes selected by EC (EC50) are either in CGC, OMIM, or NCG. The Venn diagram displays their distribution among the three databases. Of the remaining 20 genes, 10 have been discovered in GWAS studies (indicated by asterisk). (**b**) EC50 genes identified by the 10 independent classifiers. (**c**) Mapping of EC50 genes and enriched signalling pathways onto an FLN as explained in the text. Only links with weights greater than 0.1 are retained.

The average performance statistics for ovarian cancer were comparable to those of breast cancer, with TP = 0.70, TN = 0.97. Again, the specificity is uninformative. Of the EC50 genes, 30 are annotated in either CGC, OMIM, or NCG (Fig. 4a), giving a PPV rate of 0.6.

Ten of the remaining 20 (marked with an asterisk) have cancer related records in either the GWAS Catalog or GAD. The remaining 10 – *FYN, PRKCQ, MAPK3, EIF2AK3, ULK4, PRKCD, PRKD3, MAP4K3, MAST2, STK10* – are considered predictions (Fig. 4b).

A comparison of EC50 genes from breast and ovarian cancers indicates that 12 occur in both cancer types (Supplementary Table S1). Of these, 11 are identified by CGC, OMIM or NCG, as being present in at least 1 other cancer type. One gene, *PRKCQ*, is predicted to be present in both, and is not listed in any public databases. The biological implications of this finding are elaborated in the Discussion. In total, 34 of the EC50 breast cancer genes, and 30 of the EC50 ovarian cancer genes are in either CGC, OMIM or NCG and consequently occur in more than one cancer (Figs. 2a and 4a). More specifically, 16 of the 34, and 18 of the 30 are found in at least 2 other cancer types. Consequently, in keeping with the growing consensus^17^, most of our predicted genes are not tissue specific.

#### Intergenic relations

As with breast cancer, we searched for KEGG pathways that are statistically enriched in cancer drivers, and found 19 (*FDR* < 0.01) such pathways (Table 3). Figure 4c is a VisANT display of the relation between the three most significantly enriched signaling pathways: ErbB, Chemokine and Neurotrophin.

#### Predictive performance compared to individual classifiers

**Figure 5:**
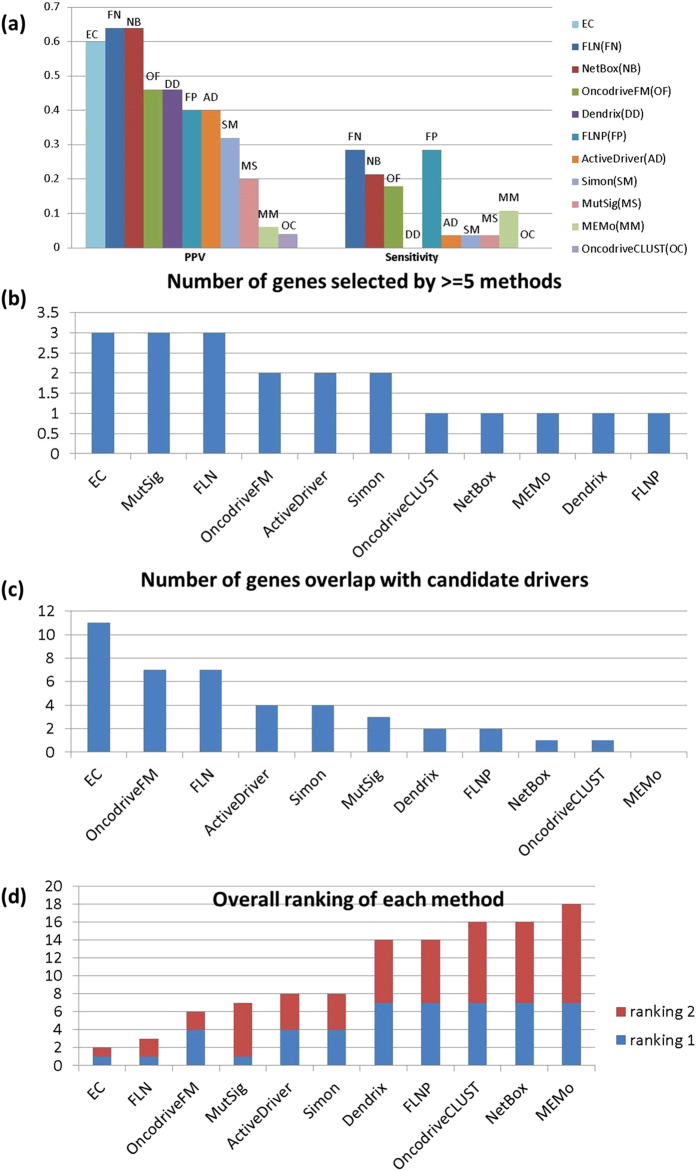
Comparison of performance metrics for the ensemble classifier and single feature classifiers for ovarian cancer. (**a**) Sensitivity and PPV for each of the methods. (**b**) The number of genes in Top 50 that are identified by at least 5 methods. (**c**) The number of genes in Top 50 that are annotated in two ovarian cancer studies. No genes can be overlapped with these two studies in MEMo. (**d**) Overall ranking of each method based on the sum of rankings in (**b**) and (**c**).

The surrogate sensitivities of the individual methods, ranged from 4% to 29%. The PPVs ranged from 4% to 64% (average 36.2%). The PPV of 64%, slightly higher than that of EC, was achieved by the FLN and NetBox (Fig. 5a).

Just as with breast cancer, we compared the EC50 genes with the Top 50 genes selected by each of the 10 independent classifiers, using criteria (i) and (ii). The cancer genes were taken from two ovarian cancer studies^27^,^35^ that include 178 candidate drivers. EC identifies 3 genes that are classified as candidate drivers by at least 5 methods (Fig. 5b) and 11 genes that overlap with existing candidates (Fig. 5c), giving it the highest overall rank (Fig. 5d). The results are consistent with those obtained for breast cancer; an integrated procedure is unique in performing well against both criteria. This result along with the results for breast cancer adds another dimension to the evidence for increased stability of EC. It not only performs at or near the top of the list when assessed against the individual methods, but does so for both cancer types.

## Discussion

For breast cancer, of the six predicted candidates (*PRKCQ, ARAF, MAPK14, BRMS1, CDC42BPA, SP3*), three (*PRKCQ, ARAF, MAPK14*) are members of at least one KEGG cancer relevant pathway. *PRKCQ* is especially intriguing. It is a member of the protein kinase C (PKC) family, and ranks sixth in the EC50 list. Equally importantly, like all PKC isoforms, its C1 domain binds phorbol esters, a class of tumor promoters. *PRKCQ* signaling regulates the accumulation of the oncogenic transcription factor Fra-1 which is overexpressed in ER negative breast cancer cells^36^. The location of *PRKCQ* on the FLN lends weight to its importance as a driver. In particular, it is directly linked to 34 other driver candidates in EC50, including *PTEN, MAPK8, CDKN1B, PRK3R1*, which are well known drivers.

Although our analysis indicates that *PRKCQ* is a prominent candidates, it is missed by 9/10 individual classifiers (Fig. 2b). It is perhaps noteworthy that it only mutates in 7 of TCGA breast tumors samples, with 6 non-silent mutations and 1 silent mutation. Among these 6 non-silent mutations, there is only 1 nonsense mutation with high impact on protein sequence, the other 5 are missense mutations that have little effects. Its low mutation rate (6/778 = 0.0078) might also contribute to the fact that it is only predicted to be significant (*P* = 0.02) by OncodriveFM, and undetectable by the 9 methods. This specific result illustrates our general finding that the sensitivity of EC is considerably greater than that of the methods used individually.

Other candidates are *ARAF* and *BRMS1*. The former is a proto-oncogene that regulates cell growth, development and differentiation and is involved in focal structural events in breast cancer^37^. *BRMS1* has a posterior probability *Prb* of 0.87 (*Prb*, a measure of distance from the decision boundary, which is at *Prb* = 0.5, see Methods), and is identified by both MutSig and ActiveDriver (Fig. 2b). There is some evidence that it suppresses metastatic breast cancer and is a potential inhibitor of tumor progression^38^. *BRMS1* promoter methylation was evaluated as a prognostic biomarker in primary breast tumors and a subset of corresponding circulating tumor cells^39^.

Figure 2c shows EC50 genes and enriched signaling pathways mapped onto a functional linkage network (FLN)^12^. The FLN has the property that neighboring nodes (genes) are functionally related, as indicated by evidence weighted links. The enrichment of the three signaling pathways (Fig. 2c) -- ErbB, Jak-STAT, TGF-beta -- is not surprising, but confirmatory: they have been widely discussed in the breast cancer literature (e.g.^16^,^40^,^41^). Perhaps of greater interest is that they strongly overlap functionally, sharing a number of genes. More specifically, our newly predicted gene *ARAF* is in the ErbB pathway. Equally suggestive is that *ARAF* has 29 strong functional connections in the FLN with other genes in EC50, and that it is tightly linked with a number of oncogenes or tumor suppressor genes including *ERBB3, JAK2, PTEN, PIK3R1, MAP3K1*.

The identification of the cellular processes pathways -- Cell cycle, Adherens junction, and Focal adhesion – is also a confirmatory result. They have all been implicated in breast cancer by previous studies^42^,^43^,^44^.

The remaining 11 enriched pathways are organismal system (immune system, endocrine system, nervous system) related. Standish *et al*^45^ have demonstrated that activation of immune response pathways; specifically, the T cell receptor^46^ plays an important reactive role in breast cancer by suppressing cell proliferation and tumor growth. On the other hand, the neurotrophin signaling pathway, which has been studied primarily in the central nervous system, may be a driver, rather than a reactive breast cancer pathway^47^,^48^. There is also one newly predicted gene, *MAPK14*, in the immune/nervous/endocrine system pathways that appears to be causal. It is involved in 29 pathways according to KEGG and linked to 39 of the Top 50 genes in the FLN, interacts strongly with other oncogenes or tumor suppressor genes, including *ERBB3, JAK2, PIK3R1, PTEN*. It may have a role as an integration point for multiple biochemical signals, and are involved in a wide variety of cellular processes such as proliferation, differentiation, transcription regulation and development.

For ovarian cancer, thirty of the EC50 genes are annotated in either CGC, OMIM or NCG, 10 are in genome-wide association study, and another 10 are predictions. We focus on the 10 predictions. First, from a purely statistical view, *FYN*, which has the highest posterior probability (*Prb* = 0.95) of the predicted genes, would seem to be the strongest, or among the strongest candidates. It also participates as a member of the driver modules identified by FLN, NetBox and FLNP (Fig. 4b). The strong statistical results find some support in biology. *FYN* encodes a membrane-associated tyrosine kinase that has been implicated in the control of cell growth. It is a Ras induced src family kinase that is overexpressed in a large number of cancers^49^.

*PRKCD* and *PRKD3* belong to protein kinase C (PKC) family, whose members also serve as major receptors for phorbol esters, a class of tumor promoters. The family of protein kinases includes many oncogenes and growth factor receptors, some of which have been linked to the pathogenesis and progression of breast cancer^50^.

The FLN provides additional insight. *PRKD3* has 40 connections with other EC50 genes in the FLN, including tumor suppressor genes, and oncogenes such as *TP53, JAK2, RAF1*. *PRKD3* was found to interact with *HDAC1* in prostate cancer by suppressing its expression and decreasing its binding to the uPA promotor^51^, interestingly, *HDAC1* is well known to deacetylates *p53* and modulates its effect on cell growth and apoptosis, indicating there might be some undiscovered relations between *PRKD3* and *p53*.

It is noteworthy that *PRKCD*, *PRKD3*, *MAP4K3* are novel findings that can’t be identified by any of the 10 methods (Fig. 4b), although they are also highly plausible based on what we know about the physiology of the processes they are involved in. These genes provide an especially informative contrast between the outcomes of integration and independent classifiers. In particular EC identifies them as strong candidates, whereas none of the classifiers used independently identify them. As an example, *PRKD3* only mutates in 3 ovarian tumors, with a mutation rate as low as 0.0095 (3/316 = 0.0095), and all 3 are missense mutations that have mild impact on protein structure. Since *PRKD3* very rarely mutates in ovarian cancer, it is difficult to detect by the individual methods.

Of the 19 KEGG pathways that are enriched in ovarian cancer, 14 overlap with enriched pathways in breast cancer. Some of the pathways that appear to be ovarian cancer specific such as MAPK signaling and VEGF signaling are generally altered tumors. Their lack of enrichment in the breast cancer EC50 suggests that they are likely false negatives, possibly reflecting stage-related biases in the cancer samples, compounded by the small number of genes that we are considering.

Four predicted genes are included in these 19 pathways (shown in bold face in Table 3). It is interesting that both of *MAPK3* and *MAP4K3* are members of mitogen-activated protein (MAP) kinase family. Relations between MAP kinase family and ovarian cancer have been discussed broadly before^52^,^53^: it is perhaps not surprising, but nevertheless supportive of the method, that the *MAPK3* (17 pathways), and *MAP4K3* (1 pathway) system is enriched in EC50 genes.

Several factors may impact our results, including the proper selection of training sets, and limitations of sample size.

For the positive gene set, we manually searched both CGC and OMIM by keywords for a particular cancer. Undoubtedly these two databases are incomplete, but they are the most thorough catalog of driver genes currently available. Due to our limited knowledge of cancer or mistakes during sequencing, the classifier built on our selected positive and negative sets will not be perfect. We have, however, reduced the effect of noise on the training set by imposing a stringent condition on acceptance, as described in methods.

Stringency in choosing data sets of course leads to a potential problem of an overly limited training set. We approached this by choosing to select an ensemble classifier, DECORATE, specifically designed to address the problem of limited data. As discussed under “Methods”, DECORATE is designed to iteratively generate artificial training examples so that an effective diverse committee could be created. Computational experiments^31^ have demonstrated that DECORATE work effectively by achieving higher accuracy than other methods, especially when training the set is small.

## Methods

### Individual classifiers

We identify 10 methods by screening the literature and select those that are publicly available, and that provide the data required for execution. For example, we omit MuSiC^6^ because the binary version of sequence alignment data (.bam) which is a required input file, is not open access.

We first implement each algorithm separately, to obtain a matrix *G* of driver candidates, where the element *g*_*ij*_ is 1 if algorithm *j* classifies gene *i* as a driver, and 0, otherwise. Here *j* runs from 1 to 10 and *i* labels the genes that are predicted by at least one algorithm (see Supplementary Table S1). For those classifiers requiring explicit mutation data (OncodriveFM, OncodriveCLUST, MutSig, MEMo, Dendrix, ActiveDriver, Simon, NetBox), we use the Cancer Genome Atlas (TCGA) breast cancer^26^ data set (.maf), which includes 52,164 somatic mutations identified in 17,042 genes from 778 breast cancer (BRCA) patients; and the TCGA ovarian cancer data^27^, which includes 19,356 somatic mutations in 9,968 genes from 316 ovarian (OV) cancer patients.

### Training and testing

The positive sets are obtained by searching keywords of breast cancer or ovarian cancer in both CGC ( http://cancer.sanger.ac.uk/cancergenome/projects/census/) and OMIM ( http://www.omim.org/), giving 37 positive genes for breast cancer, and 27 for ovarian cancer. Unfortunately, there is no gold standard for a negative set. However, two key characteristics of drivers--their distributions across cancer types, and their frequencies of occurrence across large sample sets -- can help us inform the selection of negatives. We assume that a gene is unlikely to be a driver if (i) it is mutated no more than once across all samples, 1/778 for breast cancer and 1/316 for ovarian cancer -- and (ii) it has no causally implicated mutations in other cancer types included in CGC, OMIM and NCG. We expect that the resulting set of 3943 and 4344 for breast and ovarian cancer, respectively, will have a low frequency of drivers. The primary effects of contamination of the negative set with drivers, will be that some of the predictions classified as false positives, will in fact be actual positives; i.e. our FP rate should be an upper bound.

The large set of negatives is expected, since most mutated genes will be passengers. However, the result is a highly unbalanced training set. This problem is moderated by repeatedly selecting a random sample of 37 genes from the 3943 negatives in breast cancer (or 27 of the 4344 negatives in ovarian cancer), and using it together with the positives to repeatedly train the classifier. The random selections are done through an undersampling method SpreadSubsample in weka^54^ to balance positives and negatives by setting a parameter distribution spread to 1. We repeat these *trials* 50 times to obtain 50 different training sets. Each training set is used with DECORATE and the results are averaged to obtain a posterior probability (Prb), on the basis of which an assignment is made.

Performance measures for EC are estimated by 10-fold cross validation. During training in each of the 50 trials, 10% of the genes (positives plus negatives) are set aside, and used to determine the true positive and true negative rates (sensitivity and specificity). The overall performance is assessed by averaging the performance over the 50 trials.

For each method we compute the PPV on the top 50 breast cancer predictions (after excluding any overlap with our positive training set) by testing overlap of top 50 with 2191 genes annotated as cancer related in either CGC, OMIM or NCG, but excluding breast cancer. We obtained a similar list of 2201 genes for evaluation of ovarian cancer prediction. The PPV is then the number of such genes occurring in the Top 50 divided by the number of calls, which is 50.

We evaluated the sensitivities and specifies for the 10 publicly available methods using 37 and 27 positives (P) respectively for breast and ovarian cancer, and samples of the same numbers for the negatives (N). This was done in order to keep the estimates for the 10 public classifiers consistent with the numbers used for EC. The sensitivity was then the fraction of P that are true positives in Top 50, and the specificity, the fraction of N that are true negatives outside of Top 50. Because the number of calls is so small, the allocation of a negative gene to the list almost never occurs. Hence the specificity is essentially 1.

### Screening

We download genes from ftp://ftp.ncbi.nlm.nih.gov/gene/DATA/GENE_INFO/Mammalia/ on March 2014, retaining the 20,624 genes annotated as protein-coding. The ensemble classifier is applied to the genes that remained after excluding the training sets.

### Ensemble classifier (EC)

As a minimalist approach, we use each method as a feature; i.e. we assign a 10-dimensional feature vector to each gene (Table 1). When a vector for a gene is incomplete, missing elements are assigned a value of 1 for gene methods (OncodriveFM, OncodriveCLUST, MutSig, ActiveDriver, Simon) based on *P*-values, or 0 for module methods (FLN, NetBox, MEMo, Dendrix, FLNP) based on linkage weights. Consequently, each gene is represented by a point in a 10 dimensional space.

We create ensembles of training sets using DECORATE (Diverse Ensemble Creation by Oppositional Relabeling of Artificial Training Examples)^31^, which is available on the Weka workbench^54^. The DECORATE ensemble classification model is used with the following parameters: artificialSize = 1 (the number of artificial examples added to the original training set, specified as a fraction of training data), desiredSize = 15 (the pre-defined number of ensemble classifiers in Decorate), numIteration = 50 (the maximum number of iterations to build an ensemble). The final classification is determined by using the average posterior probabilities of four base classifiers: NaiveBayes^55^, Sequential Minimal Optimization (SMO) algorithm for training a support vector classifier^56^, C4.5 decision tree^57^, and forest of random trees^58^.

We choose DECORATE because there is some evidence^31^ that for small training sets, it achieves higher accuracy than bagging^59^, or boosting^60^. DECORATE is a meta-learner classification algorithm that works on a base learner to build an effective diverse committee. It randomly generates new artificial examples in the training set by picking data points from an approximation of the training-data distribution.

## Conclusions

We developed and evaluated a principled approach to the integration of 10 driver gene/module identification methods. We found that its performance is superior to that of methods used independently, and that its reliability is more stable. The ensemble classifier identified a number of genes that are currently unrecognized as cancer related, but whose biological properties and other evidence suggest that they can reasonably be expected to play a role in cancer physiology.

## Additional Information

**How to cite this article**: Liu, Y. *et al.* Evaluation and integration of cancer gene classifiers: identification and ranking of plausible drivers. *Sci. Rep.* **5**, 10204; doi: 10.1038/srep10204 (2015).

## Supplementary Material

Supplementary Information

Supplementary Information

## Figures and Tables

**Table 1:** Summary of 10 driver gene/module identification methods.

**Method**	**How it works**	**Feature**
OncodriveFM^9^	Computes a metric of functional impact using three well-known methods (SIFT, PolyPhen2 and MutationAssessor) and assesses how the functional impact of variants found in a gene across several tumor samples deviates from a null distribution.	Uses *P*-value, which indicates whether variants within a gene are significantly accumulated with high functional impact.
OncodriveCLUST^10^	Identifies genes whose mutations tend to cluster in particular location on the protein.	Uses *P*-value, which measures the significance of gene clustering score compared with a background model that assess only silent mutations.
MutSig^7^	Estimates the background mutation rate for each gene–patient–category combination based on the observed silent mutations in the gene and non-coding mutations in the surrounding regions.	Uses *P*-value, which is determined by testing whether the observed mutations in a gene significantly exceed the expected counts based on a background model.
ActiveDriver^11^	The method is based on a logistic regression strategy and identifies 22ignalling sites in proteins that involve unexpectedly many (or few) sequence variants considering the general variability of the protein, disordered and ordered regions, density of 22ignalling-related residues (such as phosphosites), and proximity of variants/mutations to 23ignalling residues.	Uses *P*-value, which indicates statistically unexpected mutated in protein phosphorylation sites or protein kinase domains.
Simon^8^	Accounts for the functional impact of mutations on proteins, variation in background mutation rate among tumors and the redundancy of the genetic code.	Uses *P*-value, which indicates genes whose mutation rate is significantly above background.
FLN^12^	Count connections of a gene with known cancer related genes based on FLN and provide Top 100 driver genes that with maximum connections.	Uses average weights (weights are obtained from FLN) between target gene and all Top 100 genes.
NetBox^13^	Identify driver module by maximizing modularity based on Human Interaction Network (HIN).	Uses total number of links between target gene and all genes interior to the module based on HIN. Target genes exterior to the module are assigned a weight of 1; interior genes are assigned a weight of 2.
MEMo^14^	Identify network modules whose members are recurrently altered across a set of tumor samples, are known to or are likely to participate in the same biological process and are mutually exclusive.	Uses total number of links between target gene and all genes interior to the module based on HIN. Exterior and interior genes are weighted 1 and 2, respectively.
Dendrix^15^	Finds sets of genes, domains, or nucleotides whose mutations exhibit both high coverage and high exclusivity in the analysed samples.	Uses total number of links between target gene and all genes interior to the module based on HIN. Same weight as above.
FLNP (Huang *et al.*, submitted)	Identify driver module by maximizing modularity based on Functional Linkage Network (FLN).	Uses average weights (weights are obtained from FLN) between target gene and all genes interior to the module.

This table describes the 10 methods that we use to do the integration, including the name of the method, how it works, how we use it as a feature.

**Table 2:** KEGG pathways enriched in breast cancer using DAVID (*FDR* < 0.01).

**Pathway**	**Count**	**Genes**	***P*****-value**	***FDR***
ErbB signaling	12	*CDKN1B, GRB2, ERBB3, PIK3CB, JUN, **ARAF**, MAPK8, SHC1, RPS6KB1, PAK1, ABL1, PIK3R1*	3.0E-11	7.4E-10
Neurotrophin signaling	12	*GRB2, PIK3CB, MAP3K1, **MAPK14**, JUN, NFKBIA, NFKB1, MAPK8, SHC1, ABL1, IRS1, PIK3R1*	1.5E-9	2.2E-8
T cell receptor signaling	11	***PRKCQ**, GRB2, PIK3CB, **MAPK14**, JUN, IFNG, NFKBIA, NFKB1, PAK1, PIK3R1, IL2*	6.1E-9	6.6E-8
Jak-STAT signaling	10	*TYK2, GRB2, PIK3CB, IL6ST, IFNG, CREBBP, JAK2, STAT3, PIK3R1, IL2*	2.2E-6	1.6E-5
Cell cycle	9	*E2F1, CDKN1B, HDAC2, HDAC1, CREBBP, SMAD4, SMAD3, SMAD2, ABL1*	4.2E-6	2.9E-5
Toll-like receptor signaling	8	*PIK3CB, **MAPK14**, JUN, NFKBIA, NFKB1, MAPK8, TLR4, PIK3R1*	1.1E-5	6.2E-5
Adipocytokine signaling	7	***PRKCQ**, NFKBIA, NFKB1, MAPK8, JAK2, IRS1, STAT3*	1.1E-5	6.0E-5
B cell receptor signaling	7	*GRB2, PIK3CB, JUN, NFKBIA, NFKB1, PIK3R1, BTK*	2.2E-5	1.0E-4
TGF-beta signaling	7	*SP1, IFNG, CREBBP, SMAD4, SMAD3, SMAD2, RPS6KB1*	5.1E-5	2.1E-4
Insulin signaling	8	*GRB2, PIK3CB, **ARAF**, MAPK8, SHC1, RPS6KB1, IRS1, PIK3R1*	7.1E-5	2.8E-4
Chemokine signaling	9	*GRB2, PIK3CB, NFKBIA, NFKB1, JAK2, SHC1, PAK1, STAT3, PIK3R1*	8.1E-5	3.0E-4
Fc epsilon RI signaling	6	*GRB2, PIK3CB, **MAPK14**, MAPK8, PIK3R1, BTK*	3.2E-4	1.1E-3
Natural killer cell mediated cytotoxicity	7	*GRB2, PIK3CB, **ARAF**, IFNG, SHC1, PAK1, PIK3R1*	5.3E-4	1.7E-3
Focal adhesion	8	*GRB2, PIK3CB, JUN, MAPK8, SHC1, PAK1, PTEN, PIK3R1*	8.3E-4	2.4E-3
GnRH signaling	6	*MAP3K4, GRB2, MAP3K1, **MAPK14**, JUN, MAPK8*	9.3E-4	2.6E-3
RIG-I-like receptor signaling	5	*MAP3K1, **MAPK14**, NFKBIA, NFKB1, MAPK8*	2.2E-3	5.8E-3
Adherens junction	5	*CREBBP, SMAD4, SMAD3, SMAD2, MLLT4*	2.9E-3	7.6E-3

This table shows enriched KEGG pathways in breast cancer (*FDR* < 0.01), with *FDR* ascending order. The second and third columns are the number and names of the Top 50 genes in a given enriched pathway. Bold face indicates that the gene is newly predicted by EC, i.e. it is not identified as breast cancer related in any of the databases.

**Table 3:** KEGG pathways enriched in ovarian cancer using DAVID (*FDR* < 0.01).

**Pathway**	**Count**	**Genes**	***P*****-value**	***FDR***
ErbB signaling pathway	11	*PTK2, MAP2K1, GRB2, JUN, SOS1, **MAPK3**, RAF1, SHC1, ABL1, MYC, SRC*	9.2E-10	2.1E-8
Neurotrophin signaling	12	*NTRK3, MAP2K1, GRB2, JUN, NTRK1, SOS1, **MAPK3**, TP53, RAF1, SHC1, ABL1, **PRKCD***	2.4E-9	4.1E-8
Chemokine signaling	13	*MAP2K1, GRB2, RAF1, STAT1, VAV2, STAT3, PTK2, PTK2B, SOS1, **MAPK3**, JAK2, SHC1, JAK3*	5.9E-9	6.7E-8
Focal adhesion	13	*MAP2K1, GRB2, MET, RAF1, VAV2, SRC, PTK2, **FYN**, SOS1, JUN, **MAPK3**, PDGFRB, SHC1*	2.4E-8	2.3E-7
Natural killer cell mediated cytotoxicity	10	*MAP2K1, GRB2, PTK2B, **FYN**, SOS1, LCK, **MAPK3**, RAF1, SHC1, VAV2*	5.5E-8	3.7E-7
Cell cycle	10	*CREBBP, TP53, PRKDC, SMAD3, SMAD2, RB1, ABL1, MYC, CDK2, ATM*	4.5E-7	2.8E-6
Adherens junction	8	***FYN**, CREBBP, MET, **MAPK3**, SMAD3, SMAD2, INSR, SRC*	1.7E-6	8.6E-6
T cell receptor signaling	9	*MAP2K1, GRB2, **FYN**, JUN, SOS1, LCK, **MAPK3**, RAF1, VAV2*	2.2E-6	1.1E-5
GnRH signaling	8	*MAP2K1, GRB2, PTK2B, JUN, SOS1, **MAPK3**, RAF1, SRC*	1.1E-5	4.1E-5
Jak-STAT signaling	9	*GRB2, SOS1, CREBBP, JAK1, JAK2, JAK3, STAT1, MYC, STAT3*	1.5E-5	5.3E-5
B cell receptor signaling	7	*MAP2K1, GRB2, JUN, SOS1, **MAPK3**, RAF1, VAV2*	1.9E-5	6.1E-5
Fc epsilon RI signaling	7	*MAP2K1, GRB2, **FYN**, SOS1, **MAPK3**, RAF1, VAV2*	2.4E-5	7.2E-5
MAPK signaling	11	***MAP4K3**, MAP2K1, GRB2, JUN, NTRK1, SOS1, MAPK3, TP53, PDGFRB, RAF1, MYC*	4.8E-5	1.4E-4
Gap junction	7	*MAP2K1, GRB2, SOS1, **MAPK3**, PDGFRB, RAF1, SRC*	9.2E-5	2.5E-4
TGF-beta signaling	6	*SP1, CREBBP, **MAPK3**, SMAD3, SMAD2, MYC*	4.5E-4	1.1E-3
Insulin signaling	7	*MAP2K1, GRB2, SOS1, **MAPK3**, RAF1, SHC1, INSR*	5.5E-4	1.3E-3
Axon guidance	7	*PTK2, **FYN**, MET, **MAPK3**, FES, ABL1, CDK5*	6.0E-4	1.4E-3
Dorso-ventral axis formation	4	*MAP2K1, GRB2, SOS1, **MAPK3***	8.6E-4	1.9E-3
VEGF signaling	5	*PTK2, MAP2K1, **MAPK3**, RAF1, SRC*	2.6E-3	5.7E-3

This table shows enriched KEGG pathways in ovarian cancer (*FDR* < 0.01), with *FDR* ascending order. The second and third columns are the number and names of the Top 50 genes in a given enriched pathway. Bold face indicates that the gene is newly predicted by EC.
