# Supplementary material for: Evaluation and integration of cancer gene classifiers: identification and ranking of plausible drivers
Source: Sci Rep. 2015 May 11;5:10204. doi: 10.1038/srep10204 (PMC4650817; doi:10.1038/srep10204)
Supplement: Supplementary Information [file srep10204-s1.pdf]

Supplementary Table Title Page

Evaluation and integration of cancer gene classifiers: identification and ranking of plausible drivers

Yang Liu[1], Feng Tian[1], Zhenjun Hu[1], Charles DeLisi[1*]

1 Bioinformatics Graduate Program, and Department of Biomedical Engineering, Boston University, 24 Cummington Mall, Boston, MA 02215, USA

Charles DeLisi: charlesdelisi@gmail.com * Corresponding author

Supplementary Table S1 –Gene candidates identified by different methods.

This .xls file shows cancer gene candidates identified in breast/ovarian cancer by each of the 10 individual classifiers and by EC respectively.
